# Beta-1,4-galactosyltransferase-3 deficiency suppresses the growth of immunogenic tumors in mice

**DOI:** 10.3389/fimmu.2023.1272537

**Published:** 2023-10-09

**Authors:** Heng Wei, Chie Naruse, Daisuke Takakura, Kazushi Sugihara, Xuchi Pan, Aki Ikeda, Nana Kawasaki, Masahide Asano

**Affiliations:** ^1^ Institute of Laboratory Animals, Graduate School of Medicine, Kyoto University, Kyoto, Japan; ^2^ Graduate School of Medical Life Science, Yokohama City University, Yokohama, Japan

**Keywords:** galactosyltransferase, tumor immune microenvironment, N-glycosylation, glycoproteomics, immunogenicity

## Abstract

**Background:**

Beta-1,4-galactosyltransferase-3 (B4GALT3) belongs to the family of beta-1,4-galactosyltransferases (B4GALTs) and is responsible for the transfer of UDP-galactose to terminal *N*-acetylglucosamine. B4GALT3 is differentially expressed in tumors and adjacent normal tissues, and is correlated with clinical prognosis in several cancers, including neuroblastoma, cervical cancer, and bladder cancer. However, the exact role of B4GALT3 in the tumor immune microenvironment (TIME) remains unclear. Here, we aimed to elucidate the function of B4GALT3 in the TIME.

**Methods:**

To study the functions of B4GALT3 in cancer immunity, either weakly or strongly immunogenic tumor cells were subcutaneously transplanted into wild-type (WT) and *B4galt3* knockout (KO) mice. Bone marrow transplantation and CD8^+^ T cell depletion experiments were conducted to elucidate the role of immune cells in suppressing tumor growth in *B4galt3* KO mice. The cell types and gene expression in the tumor region and infiltrating CD8^+^ T cells were analyzed using flow cytometry and RNA sequencing. *N*-glycosylated proteins from WT and *B4galt3* KO mice were compared using the liquid chromatography tandem mass spectrometry (LC-MS/MS)-based glycoproteomic approach.

**Results:**

*B4galt3* KO mice exhibited suppressed growth of strongly immunogenic tumors with a notable increase in CD8^+^ T cell infiltration within tumors. Notably, *B4galt3* deficiency led to changes in *N*-glycan modification of several proteins, including integrin alpha L (ITGAL), involved in T cell activity and proliferation. *In vitro* experiments suggested that *B4galt3* KO CD8^+^ T cells were more susceptible to activation and displayed increased downstream phosphorylation of FAK linked to ITGAL.

**Conclusion:**

Our study demonstrates that *B4galt3* deficiency can potentially boost anti-tumor immune responses, largely through enhancing the influx of CD8^+^ T cells. B4GALT3 might be suppressing cancer immunity by synthesizing the glycan structure of molecules on the CD8^+^ T cell surface, as evidenced by the changes in the glycan structure of ITGAL in immune cells. Importantly, *B4galt3* KO mice showed no adverse effects on growth, development, or reproduction, underscoring the potential of B4GALT3 as a promising and safe therapeutic target for cancer treatment.

## 1 Introduction

Glycosylation is a common form of post-translational modification of proteins in eukaryotes; it is estimated that more than 50% of all proteins are glycosylated (1), and mammalian glycan structures are estimated to be present in hundreds of species. The primary types of protein glycosylation are *N*-glycosylation and *O*-glycosylation. *N*-glycosylation occurs on residues of asparagine-X-serine/threonine (Asn-X-Ser/Thr). *O*-glycosylation is usually shorter but more structurally complex than *N*-glycosylation, and is initiated by the addition of *N*-acetylgalactosamine (GalNAc) to the serine/threonine (Ser/Thr) residues of the protein backbone.

*N*-glycosylation of glycoproteins plays an important role in the stability, degradation, solubility, and activity of proteins and has important cellular functions such as inflammatory response, immune escape, cell adhesion, protein clearance, receptor activation, endocytosis, and signal transduction (2–4). Growing evidence shows that glycosylation regulates the development, spread, invasion, metastasis, and angiogenesis of tumors (5, 6). Altered glycosylation patterns have been observed in numerous cancer cell types. A common change is the increased expression of sialylated glycan structures (7). These structures play a role in the invasion and metastasis of cancer cells by promoting adhesion to endothelial cells and inhibiting cytotoxicity mediated by immune cells (8–12). Another common change is increased expression of truncated *O*-glycosylation, which is associated with increased tumor invasion and poor prognosis (13, 14). These findings suggest that glycosylation is involved in many aspects of tumorigenesis, and studies on glycosylation are essential to elucidate disease mechanisms.

Beta-1,4-galactosyltransferase (B4GALT), a family that is majorly associated with galactosylation, transfers UDP-galactose to terminal *N*-acetylglucosamine. Numerous studies have identified associations between B4GALT family members and the proliferation, metastasis, and prognosis of various tumors. *B4GALT1* is highly expressed in human glioblastoma, and its knockdown controls the growth of this cancer and affects apoptosis and autophagy (15). High expression of *B4GALT4* is associated with poor prognosis in hepatocellular carcinoma (HCC). *B4GALT4* knockdown downregulates lumican secretion and suppresses the expression of microtubule and spindle assembly-associated oncogenes PLK1 and RHAMM (16). B4GALT5 modulates the stemness of breast cancer cells by stabilizing FZD1 and activating Wnt/β-catenin signaling (17). A past study found that *B4GALT5* was overexpressed in human HCC tissues, which was associated with poor prognosis and tumor progression. *B4GALT5* knockdown significantly reduced HCC cell proliferation, migration, and invasion (18). B4GALT1 and B4GALT5 control the activity of hedgehog signaling and proportionally mutative expression of p-glycoprotein and MRP1 in the development of multidrug resistance in human leukemia cells (19). However, reports on the glycosylation of immune cells in the tumor microenvironment are rare. Moreover, B4GALTs and galactose-containing carbohydrates in the tumor immune microenvironment (TIME) have not been studied.

B4GALT3 is associated with prognosis and proliferation in various cancers. Chang et al. reported that high expression of B4GALT3 promotes tumor growth and migration in neuroblastoma, resulting in poor prognosis for patients (20). However, Chen et al. reported that in colorectal cancer, high expression of *B4GALT3* inhibits cell migration, invasion, and adhesion, and is negatively correlated with poorly differentiated histology, advanced stages, regional lymph node metastasis, and distant metastasis (21). Recent studies have shown that the knockdown of *B4GALT3* promotes fibroblast motility and leads to the activation of integrin beta 1 (ITGB1) *via* NF-κB signaling in fibroblasts, which promotes the development of lung metastases from HCC through the secretion of IL-6 and IL-8 (22). B4GALT3 is associated with various tumors; however, its role in the TIME is unclear. We examined the growth of various strongly or weakly immunogenic tumor cell lines transplanted into *B4galt1, B4galt3*, and *B4galt4* knockout (KO) mice. This investigation aimed to elucidate the function of B4GALT3 in the TIME, and determine its potential clinical implications for cancer therapy.

## 2 Materials and methods

### 2.1 Mice

All the mice used in this study were housed in a specific pathogen-free environment at the Institute of Laboratory Animals, Graduate School of Medicine, Kyoto University. They were maintained at 22–24°C and 50–60% humidity with a 14-h light (07:00-21:00)/10-h dark cycle. For anesthesia, a combination of three drugs was administered: medetomidine (Domitor; Meiji Seika Pharma, Tokyo, Japan) at 0.3 mg/kg body weight, midazolam (Dormicum; Astellas, Tokyo, Japan) at 4 mg/kg body weight, and butorphanol (Betolfal; Meiji Seika Pharma, Tokyo, Japan) at 5 mg/kg body weight. The final volume was adjusted using physiological saline at a dosage of 10 ml/Kg. When euthanasia was deemed necessary, it was carried out using the cervical dislocation method by technically skilled personnel to ensure a humane and ethical procedure, in line with the guidelines set by the Institute of Laboratory Animals.

### 2.2 Generation of *B4galt3* and *B4galt4* KO mice by CRISPR/Cas9 genome editing

Four-week-old female C57BL/6 mice (Japan SLC Inc, Shizuoka, Japan) were superovulated by 0.1 ml of HyperOva (KYUDO, Saga, Japan), followed by 5 IU of human chorionic gonadotropin (hCG; GONATROPIN, ASKA Pharmaceutical, Tokyo, Japan) 48 h later. The following morning, the ovum was collected for *in vitro* fertilization with the sperm of more than 12-week-old male C57BL/6 mice (Japan SLC Inc). One-cell-stage fertilized eggs were collected by washing with M2 medium (ARK Resource, Kumamoto, Japan) and maintained in KSOM medium (ARK Resource).

The target sequences of the gRNA were designed using the online tool, Konezumi (Tsukuba University). Target-specific Alt-R^TM^ CRISPR crRNA (IDT, Iowa, USA), tracrRNA (IDT), and Cas9 enzyme (IDT) were mixed and electroporated into zygotes using an electroporator (NEPA21; Nepagene, Tokyo, Japan) according to the manufacturer’s instructions. The gRNA sequences are listed in **Supplementary Table 1**. Zygotes were then transferred into the oviducts of pseudopregnant ICR female mice (Japan SLC Inc) 0.5 days post copulation. The desired mutant mice were backcrossed with C57BL/6 mice prior to the experiments.

### 2.3 Mice genotyping

Tail tips were cut from the mice and genotyped using genomic DNA PCR. The PCR products were purified using the NucleoSpin Gel and PCR Clean-up kit (Takara Bio Inc, Shiga, Japan) and analyzed by Sanger sequencing (Macrogen Japan, Tokyo, Japan). Primer sequences are presented in **Supplementary Table 1**. Heterozygous F1 mice were bred to generate homozygous F2 mice.

### 2.4 Cell lines and transplantation

Tumor cells were injected subcutaneously into 2-3 month-old anesthetized female mice using syringes fitted with 23G needles (Terumo, Tokyo, Japan), except that EO771 cells were injected into the fourth mammary fat pad. Tumor size was measured every two to three days using a digital caliper. The tumor volume was calculated as follows: volume = (length of longer axis) × (length of shorter axis)^2^ × 0.52 (23, 24). The tumor transplantation experiment had three endpoints: a tumor size of 2,000 mm^3^, weight loss of ≥ 20% from the start of the experiment, and debilitation. Details of the cell culture conditions and number of transplants are shown in **Supplementary Table 2**. All cells were cultured in a humidified incubator at 37°C with 5% CO_2_.

### 2.5 Transplantation of bone marrow cells into mice

Bone marrow cells (BMCs) were collected from thigh bones of wild-type (WT) and *B4galt3* KO donor mice post-euthanasia. The erythrocytes were lysed in RBC lysis buffer (140 mM NH_4_Cl in 17 mM Tris-HCl, pH7.2) for 5 min and the cells were washed thoroughly with Hanks’ balanced salt solution (HBSS) buffer. The recipient mice were irradiated with 9.5 Gy gamma radiation (Nordion, Ontario, Canada), and consequently died without the transplantation of BMCs. Subsequently, 2 × 10^6^ BMCs were resuspended in 100 µL HBSS buffer and transplanted from the orbital plexus into anesthetized C57BL/6 recipient mice. MC38 and EO771 cells were transplanted into recipient mice 30 days after the transplantation of BMCs.

### 2.6 CD8^+^ T cell depletion

Relative to the tumor injection day (day 0), mice were intraperitoneally injected three times with CD8^+^ T cell depleting antibody (A2102, Selleck, Houston, USA) or isotype control antibody (A2116, Selleck) on days -2 (100 µg), -1 (100 µg), and 7 (250 µg). On day 21, tail vein blood was collected and CD8^+^ T cell depletion efficiency determined using flow cytometry.

### 2.7 Preparation of splenocytes and T cell activation

To prepare splenocytes from euthanized mice, spleens were cut with scissors and crushed using a plunger in a 3.5 mm dish containing 1 mL RPMI 1640 medium, then passed through a 70 µm cell strainer to form a single-cell suspension. The erythrocytes were lysed in RBC lysis buffer for 5 min, and the cells were washed thoroughly with PBS-BSA buffer. After adjusting the cell numbers, the cells were cultured in RPMI 1640 medium supplemented with 10% FBS, GlutaMAX, and penicillin-streptomycin. T cells (1 × 10^6^) in splenocytes were activated with 1 × 10^6^ Dynabeads Mouse T-Activator CD3/CD28 (Invitrogen). Membrane proteins were isolated using a Minute Plasma Membrane Protein Isolation Kit (Invent, Plymouth, USA) according to the manufacturer’s instructions. After separation, the proteins were stored at –80°C for future glycosylation analysis.

For focal adhesion kinase (FAK) phosphorylation, CD8^+^ T cells were isolated from splenocytes using the EasySep Mouse CD8^+^ T Cell Isolation Kit (STEMCELL Technologies, Vancouver, Canada). The cells were then divided into groups and, as needed, 20 µM BIRT 377, an inhibitor for LFA-1 (FUJIFILM Wako, Tokyo, Japan) was added. CD8^+^ T cells were activated using Dynabeads Mouse T-Activator CD3/CD28 (Invitrogen). After 6-h of stimulation, cells were subjected to flow cytometry to analyze protein phosphorylation. The preparation involved staining the cell membrane with a suitable marker followed by washing the cells with PBS. In accordance with the manufacturer’s instructions, the Cyto Fast Fix/Perm buffer set (BioLegend, San Diego, USA) was used to fix and permeabilize the cells. Subsequently, the cells were stained at room temperature for 30 min and rinsed with CSB buffer solution prior to fluorescence-activated cell sorting (FACS) analysis.

### 2.8 Fluorescence-activated cell sorting

Tumor tissues from the TIME with transplanted EO771 cells were dissected from the mammary fat pad and cut into pieces with a diameter of less than 1 mm in a culture dish containing 5 ml of Dulbecco’s modified Eagle medium (DMEM). Tumor tissues were dissociated using Tissue Dissociation Reagent (BD Bioscience, New Jersey, USA) according to the manufacturer’s instructions. Summarily, the excised tissues in DMEM were transferred to a 50-mL tube containing 2X TTDR and stirred gently and frequently at 37°C for 30 min. Then, 25 mL of 1% BSA/PBS/2mM EDTA was added to stop the digestion reaction. The erythrocytes were then lysed with 1X BD Pharm Lyse (BD Biosciences) for 15 min at room temperature. Finally, cells were collected through a 70 µm filter, counted, and adjusted to a density of 1 x 10^7^/ml using cell staining buffer (PBS containing 2% FBS and 2 mM EDTA). Cells were stained using the LIVE/DEAD Fixable Far Red dead cell stain (Invitrogen), and non-specific binding was blocked with anti-mouse CD16/32 (BioLegend). The cells underwent immunostaining with the use of antibodies, as shown in **Supplementary Table 3**. The cells were analyzed using a FACSAria IIIu (BD Bioscience). Flow cytometry data were analyzed using the FlowJo software (FlowJo, Ashland, USA).

### 2.9 Tumor tissue RNA sequencing and data analysis

Tumor tissues were removed from the mice on day 23 after tumor cell transplantation and total RNA was extracted using the RNAiso Plus kit (Takara Bio Inc). cDNA library building and RNA sequencing (RNA-seq) were performed using a commercially available service (BGI, Kobe, Japan). Data analysis and visualization were performed using BGI’s in-house customized data-mining system, Dr. Tom.

### 2.10 Tumor-infiltrating CD8^+^ T cell RNA sequencing and data analysis

On day 23, after tumor transplantation, CD8^+^ T cells were isolated from EO771 tumor-bearing mice by flow cytometry. CD8^+^ T cells were suspended at 200 cells/µL in 1% PBS-BSA buffer, and 1 µL of the cell suspension was added to a specially prepared lysis buffer containing 10 mM dNTP, RNase inhibitor, and 10% Triton X-100. The samples were then centrifuged gently at 4°C and stored at –80°C. RNA extraction, cDNA library construction, and RNA-seq were performed using a commercially available service (BGI). Data analysis and visualization were performed using the Dr. Tom Data Visualization Solution.

### 2.11 Trypsin/Lys-C digestion and glycopeptide enrichment

Glycoproteins underwent a precipitation process initiated by the addition of a triple volume of cold acetone, followed by a 16-h incubation period. Centrifugation ensued at 12,000 ×g for a 10-minute duration at 4°C. The resulting precipitate was then subject to reduction with 10 mM dithiothreitol at 56°C for 30 min and subsequent alkylation using 20 mM iodoacetamide at room temperature (25°C) in dark conditions for 40 minutes. *N*-glycoproteins were broken down with 1.8 µg of a trypsin/Lys-C mixture (Promega, Wisconsin, USA) for 16-h at a steady 37°C in a continuously agitating thermomixer set at 800 rpm. The glycopeptides precipitated through the addition of a quintuple volume of cold acetone, followed by a 16-h incubation period and centrifugation at 12,000 ×*g* for 10 minutes (25). The precipitate that formed was desalted using a GL-Tip SDB (GL Science, Tokyo, Japan) and subsequently dried utilizing a SpeedVac concentrator.

### 2.12 LC/MS/MS and identification of the *N*-glycopeptides

The dried glycopeptides were reconstituted in 12 µL of 0.1% v/v formic acid, and a 4 µL aliquot of this sample was subjected to separation using an EASY-nLC 1000 system (Thermo Fisher Scientific). The separation occurred on an Acclaim PepMap100 C18 LC column (75 µm × 20 mm, 3 µm, Thermo Fisher Scientific), paired with a nano HPLC capillary column (75 µm × 120 mm, 3 µm, C18; Nikkyo Technos, Tokyo, Japan). The elution system comprised 0.1% v/v formic acid (pump A) and acetonitrile with 0.1% v/v formic acid (pump B). With a flow rate of 0.3 µL/min, glycopeptides were eluted utilizing a linear gradient from 0 to 35% B across 120 minutes.

Subsequent mass spectra were collected on a Q Exactive mass spectrometer (Thermo Fisher Scientific), with the addition of a Nanospray Flex Ion Source (Thermo Fisher Scientific), operating in positive ion mode. An Xcalibur 4.4 workstation (Thermo Fisher Scientific) facilitated the control and acquisition of MS data. We operated with a spray voltage of 1.8 kV, maintaining the capillary temperature at 250°C, and setting the S-lens RF level at 50. Full mass spectra were gathered with an *m*/z range of 350–2000 at a 70,000 resolution. We used a data-dependent acquisition method to acquire product ion mass spectra against the 20 most intense ions, working with a resolution of 17,500, normalized collision energy (NCE) of 27 and 35, and an exclusion duration of 30s. All samples underwent one analytical replicate.

The Byonic search engine version 3.11 (Protein Metrics, CA, USA), incorporated into Proteome Discoverer version 2.4 (Thermo Fisher Scientific), was employed to process raw data files. For the database search, we utilized the UniProtKB database for humans (status/2022/02) and the *N*-glycan database (mammalian; 309 entries). Search conditions included trypsin with a maximum number of missed cleavages set at 2, static modification of carbamidomethylation (C), dynamic modifications of Gln > pyroGlu (N-term Q) and oxidation (M), precursor mass tolerance of 10 ppm, fragment mass tolerance of 20 ppm, and a maximum of 2 *N*-glycosylations per peptide. Following this, all identified peptides were filtered at a false discovery rate threshold of 1% through the use of a target/decoy search strategy. The reliability of the identified peptides was evaluated using the percolator node, and only high-confidence *N*-glycoforms were processed with Microsoft Excel version 2209 (Microsoft, Washington, USA).

For the label-free quantification of *N*-glycoforms, we applied a combination of the Minora Feature Detector, Feature Mapper, and Precursor Ions Quantifier nodes in Proteome Discoverer 2.4 (Thermo Fisher Scientific). The analytical conditions included selecting unique peptides for use and the area for Precursor Quantification. The normalization mode was set according to the total peptide amount.

### 2.13 Graphical illustrations

All graphical illustrations were created using BioRender.com.

### 2.14 Statistics

Statistical analyses were performed using Prism 8 for Mac (GraphPad Software Inc, San Diego, USA). Data comparison between two conditions was performed utilizing the Student’s t-test. Unless otherwise stated, data are displayed as the mean ± standard error of the mean (SEM). All figures indicate statistical significance using these designations: *p < 0.05, **p < 0.01, ***p < 0.001, ****p < 0.0001, and ns for non-significance.

## 3 Results

### 3.1 Messenger RNA expression levels of *B4GALT3* in human pan-cancer

Recent studies have shown that B4GALT3 is associated with tumor proliferation and metastasis. We analyzed *B4GALT3* mRNA expression levels across all tumors in The Cancer Genome Atlas (TCGA) using the TIMER2.0 database (**Supplementary Figure 1A**). The results showed that *B4GALT3* was highly expressed in bladder urothelial carcinoma (BLCA), breast invasive carcinoma (BRCA), cervical squamous cell carcinoma and endocervical adenocarcinoma (CESC), cholangiocarcinoma (CHOL), colon adenocarcinoma (COAD), esophageal carcinoma (ESCA), glioblastoma multiforme (GBM), head and neck squamous cell carcinoma (HNSC), kidney renal clear cell carcinoma (KIRC), liver hepatocellular carcinoma (LIHC), lung adenocarcinoma (LUAD), lung squamous cell (LUSC), pheochromocytoma and paraganglioma (PCPG), prostate adenocarcinoma (PRAD), rectum adenocarcinoma (READ), stomach adenocarcinoma (STAD), thyroid carcinoma (THCA), uterine corpus endometrial carcinoma (UCEC), and lowly expressed in kidney chromophobe (KICH) and kidney renal papillary cell carcinoma (KIRP) compared with adjacent normal tissues. *B4GALT3* expression was higher in metastatic skin cutaneous melanoma (SKCM-metastasis) than in primary skin cutaneous melanoma (SKCM-Primary).

High expression of *B4GALT3* was associated with poor prognosis of overall survival in adenoid cystic carcinoma (ACC) (*p* = 0.003), CESC (*p* = 0.014), liver hepatocellular carcinoma (LIHC) (*p* = 0.0059), MESO (mesothelioma) (*p* = 0.02), sarcoma (SARC) (*p* = 0.044), and HNSC (*p* = 0.04) cancers based on the GEPIA2 web tool analysis (**Supplementary Figures 1B–G**). These findings reveal that high *B4GALT3* mRNA expression is associated with poor clinical survival, which merits further investigation.

### 3.2 Suppression of strongly immunogenic tumor growth in *B4galt3* KO mice

**Figure 1:**
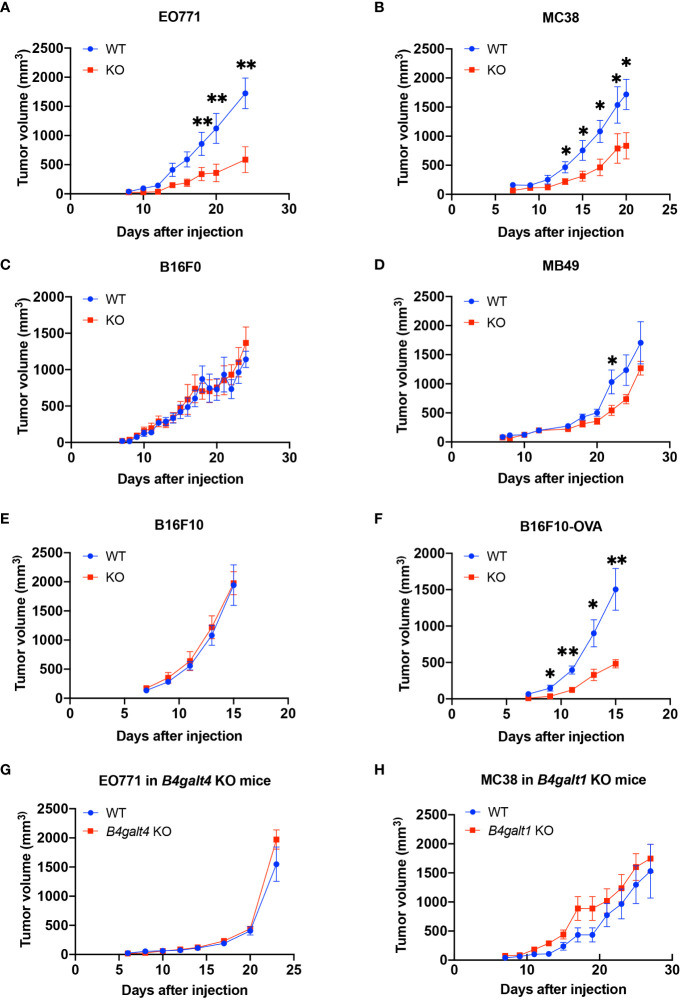
Tumor growth curves in WT (blue circles) or *B4galt3* KO mice (red squares). **(A)** EO771 cells, WT, n = 6; KO, n = 5. **(B)** MC38 cells, WT, n = 7; KO, n = 8. **(C)** B16F0 cells, WT, n = 7; KO, n = 9. **(D)** MB49 cells, WT, n = 8; KO, n = 9. **(E)** B16F10 cells, WT, n = 7; KO, n = 8. **(F)** B16F10-OVA cells, WT, n = 7; KO, n = 6. **(G)** Tumor growth curves of WT (blue circles) or *B4galt4* KO mice (red squares) inoculated with EO771 cells. WT, n = 8; KO, n = 7. **(H)** Tumor growth curves of WT (blue circles) or *B4galt1* KO mice (red squares) inoculated with MC38 cells. WT, n = 6; KO, n = 7. Mean ± SEM, *p < 0.05, **p < 0.01.

To explore the role of B4GALT3 in the TIME, we first investigated the growth of four C57BL/6 strain tumor models with varying immunogenicity: breast adenocarcinoma E0771, colon carcinoma MC38, melanoma B16F10, and bladder carcinoma MB49. Strongly immunogenic E0771 and MC38 tumors grew more slowly in *B4galt3* KO mice (**Figures 1A, B**), whereas the growth rate of weakly immunogenic B16F0 and MB49 tumors was not changed by *B4galt3* deficiency (**Figures 1C, D**). To investigate how immunogenicity influences growth kinetics, we monitored the growth of weakly immunogenic B16F10 tumors and B16F10 tumors expressing the immunogenic model antigen ovalbumin (strongly immunogenic B16F10-OVA). *B4galt3* KO mice exhibited growth suppression of B16F10-OVA tumors but not B16F10 tumors (**Figures 1E, F**).

We also examined tumor growth in *B4galt1* or *B4galt4* KO mice. *B4galt4* is most homologous to the *B4galt3* gene, and the growth of the strongly immunogenic E0771 breast cancer cell line in *B4galt4* KO mice was unaffected (**Figure 1G**). *B4galt1* is a widely studied gene in the *B4galt* gene family; however, its function in tumor immunity is unknown. We observed no difference in the growth rate of strongly immunogenic MC38 tumor cells between *B4galt1* KO and WT mice (**Figure 1H**). These findings show that only *B4galt3* deficiency can suppress the growth of strongly immunogenic tumors, whereas *B4galt1* and *B4galt4* deficiency cannot.

### 3.3 Enhancement of antitumor activity by *B4galt3* deficiency through CD8^+^ T cells

**Figure 2:**
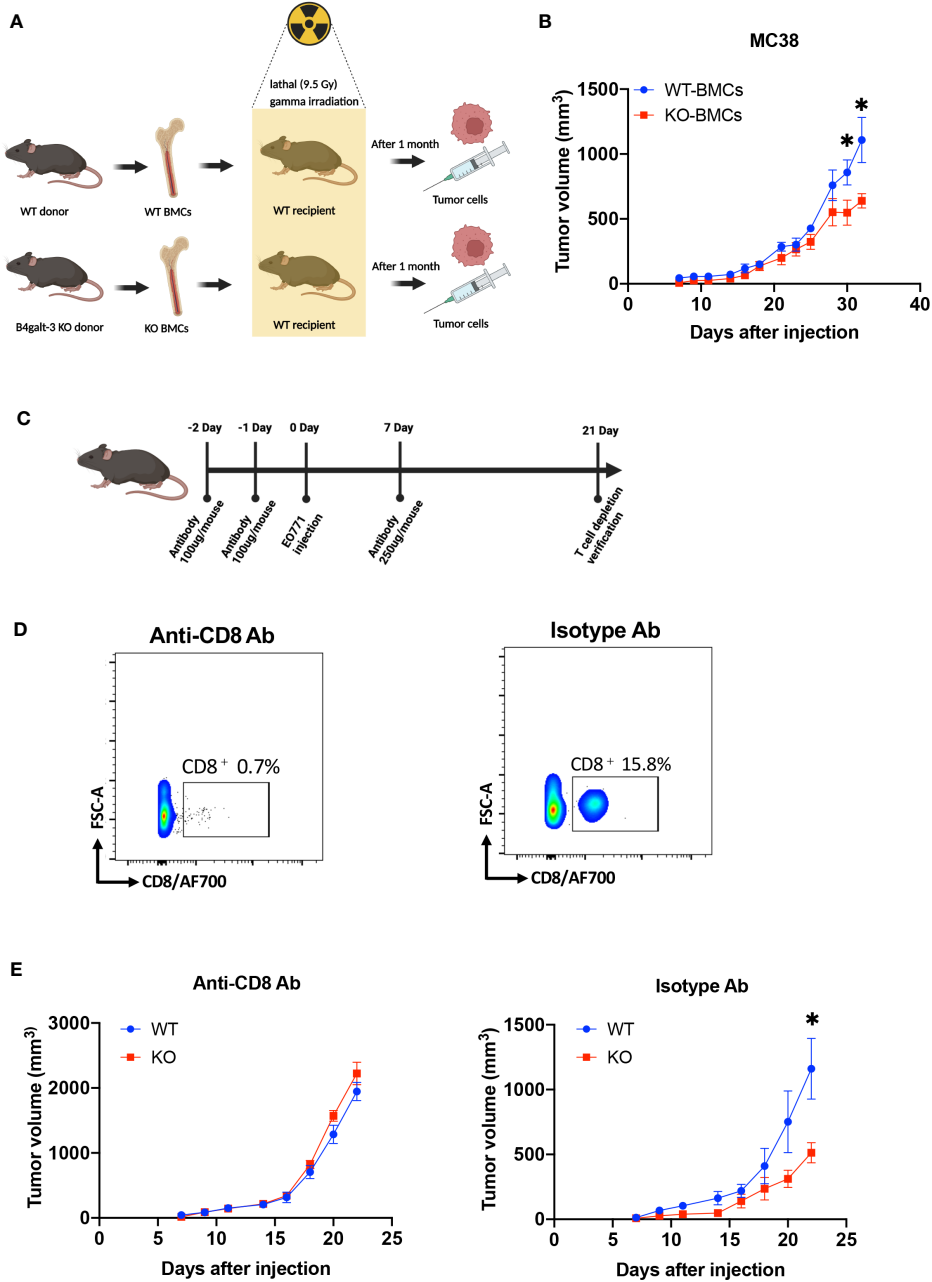
*B4galt3* deficiency suppressed tumor growth by CD8^+^ T cells. **(A)** Schematic of BMCs transplantation and MC38 cell inoculation. **(B)** Tumor growth curves in WT mice with WT BMCs transplants (n = 7, blue circles) or *B4galt3* KO BMCs (n = 8, red squares) inoculated with MC38 tumor cells. Mean ± SEM, *p < 0.05. **(C)** Schematic of CD8^+^ T cell depletion. **(D)** FACS plots of relative abundance of CD8^+^ T cells on day 21 treated with anti-CD8 antibody or isotype control antibody to deplete CD8^+^ T cells. **(E)** Tumor growth curves in WT (n = 6, blue circles) or *B4galt3* KO mice (n = 6, red squares) inoculated with EO771 tumor cells treated with anti-CD8 antibody or isotype control antibody. Mean ± SEM, *p < 0.05.

We further investigated whether B4GALT3 controls tumor growth by modulating the immune system. We performed tumor-bearing experiments using a bone marrow (BM) transplantation mouse model (**Figure 2A**). WT mice engrafted with *B4galt3* KO BM cells showed suppressed growth of strongly immunogenic MC38 tumors compared with WT mice engrafted with WT BM cells (**Figure 2B**). Therefore, B4GALT3 controls tumor growth through the immune system. Given the pivotal role of CD8^+^ T cells in anti-tumor activity, we sought to determine whether the tumor suppression observed in *B4galt3* KO is dependent on CD8^+^ T cells. To this end, we conducted experiments to deplete CD8^+^ T cells (**Figure 2C**). The CD8^+^ T cell frequency in the peripheral blood of mice injected with the CD8^+^ T cell depletion antibody was below 1%, but that of mice injected with the isotype antibody exceeded 15% (**Figure 2D**). In mice with CD8^+^ T cell depletion, there was no change in the tumor growth rate between *B4galt3* KO and WT mice (**Figure 2E**), although the tumor growth rate was suppressed in *B4galt3* KO mice with isotype antibody. These data suggest that the deletion of *B4galt3* suppresses the growth of strongly immunogenic tumors by controlling the antitumor activity of CD8^+^ T cells.

### 3.4 Characterization of gene expression in the TIME

**Figure 3:**
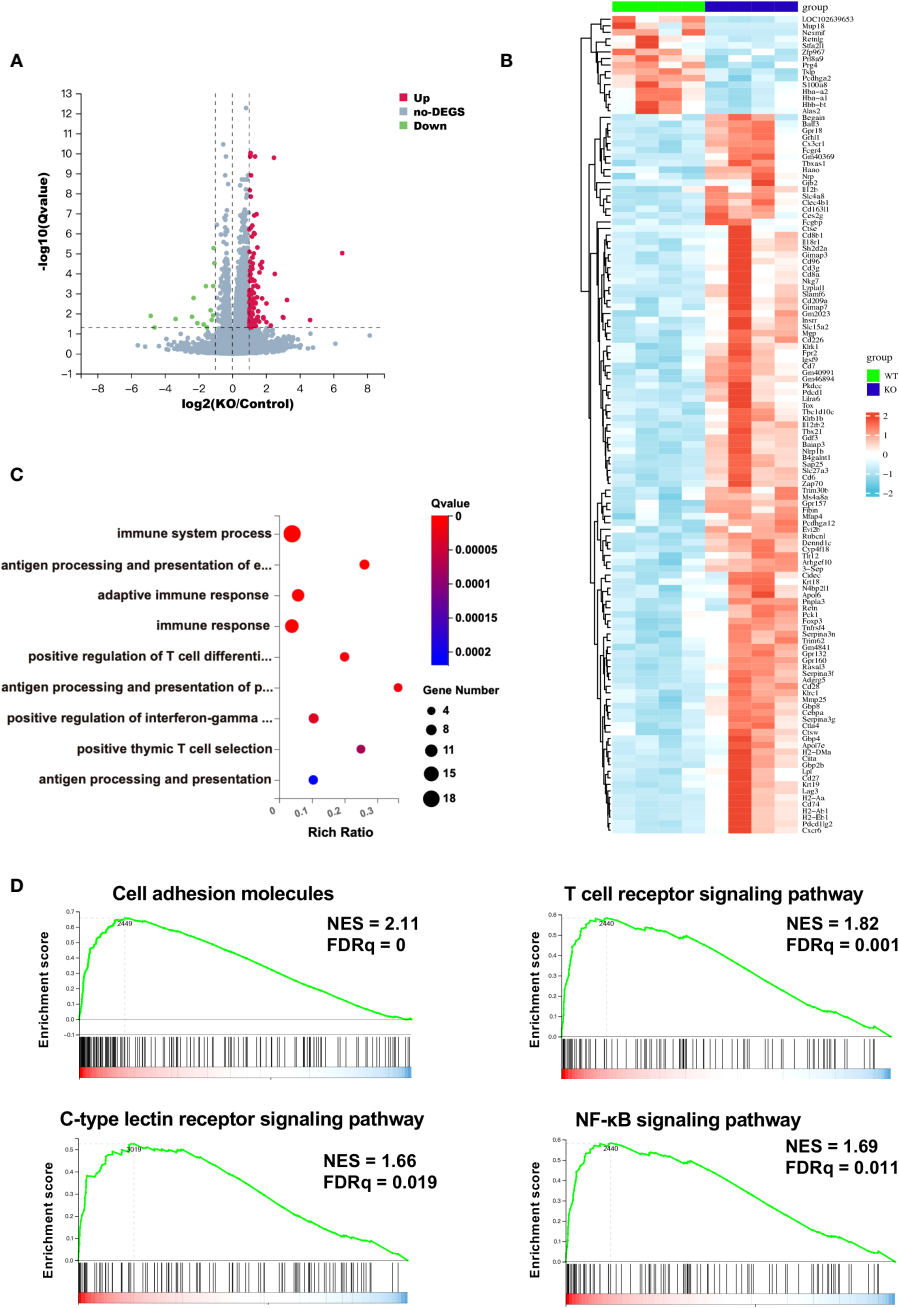
Changes in gene expression in the TIME measured by RNA-seq analysis. **(A)** Differentially expressed genes (DEGs) in *B4galt3* KO mice compared to WT mice were defined as the genes with log2 (fold change) ≥ 1 and FDR < 0.05. Red dots represent up-regulated genes, and blue dots represent down-regulated genes compared to WT mice. WT, n = 4; KO, n = 4. **(B)** Heat map of DEGs in WT mice (left side 4 rows) and *B4galt3* KO mice (right side 4 rows). **(C)** GO enrichment of up-regulated genes in *B4galt3* KO mice compared to WT mice. **(D)** GSEA of the genes associated with cell adhesion molecules, T cell receptor signaling pathway, C-type lectin receptor signaling pathway, and NF-κB signaling pathway. NES, normalized enrichment score; FDR, false discovery rate. All analyses were performed by comparing the *B4galt3* KO against WT counterparts, which served as the control group.

To further characterize the TIME of *B4galt3* deficiency, changes in gene expression were measured using RNA-seq analysis. Based on the analysis of 4 WT and 4 KO mice, 110 upregulated, and 15 downregulated differentially expressed genes (DEGs) (|log2FC| ≥ 1 and FDR ≤ 0.05) were detected (**Figure 3A**). The heat map shows the expression levels of the DEGs in each sample (**Figure 3B**). The upregulated and downregulated genes showed similar tendencies in WT and KO mice. Gene ontology (GO) analysis revealed that the DEGs were enriched in biological processes such as positive regulation of T cell differentiation and positive regulation of interferon-gamma production (**Figure 3C**). The functional classification of upregulated DEGs based on GO analysis is listed (**Supplementary Table 4**). *B4galt3* KO was found to significantly change the gene expression related to the immune system process in the TIME. To explore the mechanism of B4GALT3 function in the TIME, we analyzed our RNA-seq data using Gene Set Enrichment Analysis (GSEA) from KEGG database (**Supplementary Table 5**). *B4galt3* KO was positively correlated with cell adhesion molecules (FDRq = 0), the T cell receptor signaling pathway (FDRq = 0.001), the C-type lectin receptor signaling pathway (FDRq = 0.019), and the NF-κB signaling pathway (FDRq = 0.011) (**Figure 3D**).

### 3.5 Gene expression characteristics of tumor-infiltrating CD8^+^ T cells

To further investigate the features of *B4galt3* deficiency in the gene expression characteristics of CD8^+^ T cells in the TIME, FACS was used to sort CD8^+^ T cells from EO771 tumor-bearing mice for RNA-seq analysis (**Supplementary Figure 3A**). We identified 186 upregulated DEGs and 101 downregulated DEGs (|log2FC| ≥ 1 and FDR ≤ 0.05) (**Supplementary Figure 3B**). GO analysis revealed that the upregulated DEGs were enriched in biological processes such as regulation of cell proliferation, inflammatory response, and T cell differentiation involved in immune response (**Supplementary Figure 3C**, **Supplementary Table 6**). Interestingly, GSEA from the Wikipaths database showed that *B4galt3* deficiency was positively associated with integrin-mediated cell adhesion; however, this association was not statistically significant (**Supplementary Figure 3D**, **Supplementary Table 7**).

### 3.6 *B4galt3* deficiency enhances CD8^+^ T cell infiltration and cytotoxic activity in the TIME

**Figure 4:**
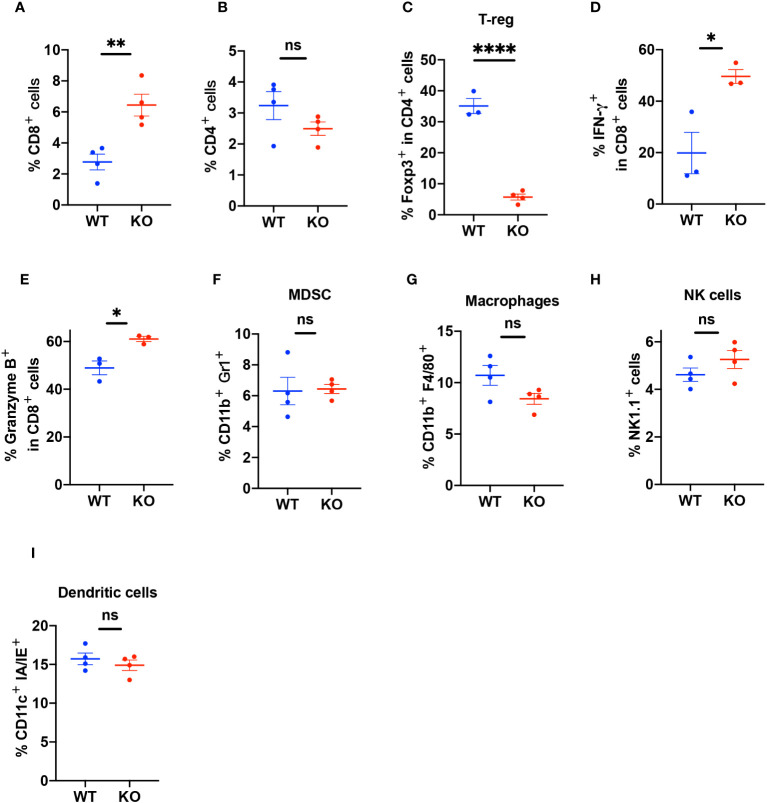
Quantification of the percentage of infiltrating immune cells in the TIME. **(A)** CD3^+^CD8^+^ T cells as a percentage of live cells in the TIME **(B)** CD3^+^CD4^+^ T cells as a percentage of live cells in the TIME. **(C)** CD3^+^CD4^+^Foxp3^+^ Tregs as a percentage of CD4^+^ T cells. **(D)** CD8^+^IFN-γ^+^ cells as a percentage of CD8^+^ T cells. **(E)** CD8^+^Granzyme B^+^ cells as a percentage of CD8^+^ T cells. **(F)** CD11b^+^Gr1^+^ (MDSC) cells as a percentage of live cells in the TIME. **(G)** CD11b^+^F4/80^+^ (macrophages) cells as a percentage of live cells in the TIME. **(H)** CD45^+^CD3^-^Nk1.1^+^ (NK cells) cells as a percentage of live cells in the TIME. **(I)** CD11c^+^ IA/IE^+^ (dendritic cells) as a percentage of live cells in the TIME. Representative data of experiments with n = 3 or 4 mice per experiment and group. Mean ± SEM, *p < 0.05, **p < 0.01, and ****p < 0.0001; ns = not significant.

To elucidate the characteristics of infiltrating immune cells, we used FACS to analyze the tumor tissues (**Supplementary Figures 4A–G**). The results showed that the TIME in the KO mice had more infiltration of CD8^+^ T cells compared to the TIME in the WT mice (**Figure 4A**) but there was no difference in CD4^+^ T cell infiltration (**Figure 4B**). The TIME of *B4galt3* KO mice showed lower levels of regulatory T cells (Tregs) (**Figure 4C**). CD8^+^ T cells expressed more interferon (IFN)-γ and granzyme B in KO mice (**Figures 4D, E**). In contrast, we did not observe significant changes in the infiltration of myeloid-derived suppressor cells (MDSC), macrophages, NK cells, or dendritic cells (**Figures 4F–I**). These results showed that *B4galt3* deficiency enhanced the infiltration and cytotoxic activity of CD8^+^ T cells in the TIME.

### 3.7 *N*-glycoproteomic analysis of *B4galt3* KO and WT immune cells

**Figure 5:**
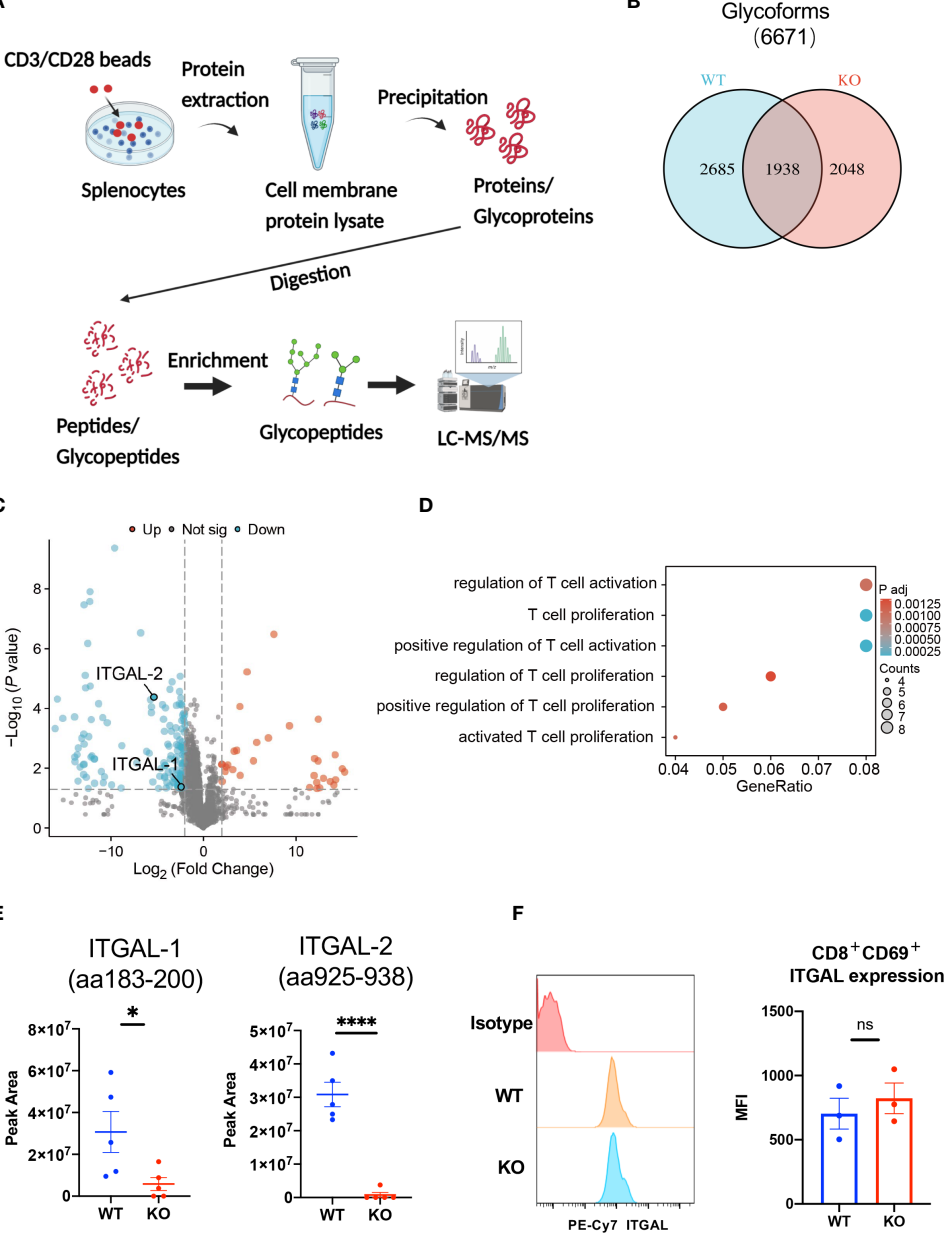
Glycoproteomic analysis of *B4galt3* KO and WT splenocytes. **(A)** Schematic of glycoproteomic analysis. **(B)** Venn diagram representation of *N*-glycoforms between WT and *B4galt3* KO splenocytes. **(C)** Volcano plot of individual *N*-glycopeptide abundance fold changes with |log2FC| ≥ 2 and *p* < 0.05 in WT and *B4galt3* KO splenocytes. Red dots represent up-regulated *N*-glycopeptides, and blue dots represent down-regulated *N*-glycopeptides in *B4galt3* KO mice compared to WT mice. **(D)** GO enrichment biological process analysis of relative abundance of *N*-glycoproteins. **(E)** Amounts of *N*-glycoforms of ITGAL-1 (aa183-200) and ITGAL-2 (aa925-938) in WT and *B4galt3* KO splenocytes. **(F)** Left panel: Representative histograms of ITGAL protein expression on the surface of CD8^+^CD69^+^ T cells. Right panel: Quantification of the mean fluorescence intensity (MFI) for ITGAL. Mean ± SEM, n = 3 mice per experiment and group. *p < 0.05 and ****p < 0.0001; ns = not significant.

To investigate the effect of *B4galt3* deficiency on protein glycosylation, protein samples were collected from the splenocytes of WT and KO mice (five mice per genotype) after activation by CD3/CD28 dynamic beads. Because the amount of protein obtained from CD8^+^ T cells infiltrated in the TIME was insufficient for glycoprotein analyses, we used stimulated spleen cells instead. Protein samples were digested with trypsin and Lys-C, and *N*-glycopeptides were enriched by acetone precipitation. A comprehensive label-free quantitative analysis was performed by liquid chromatography-tandem mass spectrometry (LC-MS/MS) at two different NCE of 27 and 35 (**Figure 5A**). Overall, 6,671 *N*-glycoforms were identified in 1,281 proteins. Among them, 2,685 *N*-glycoforms were found in the WT, 2,048 glycoforms in the *B4galt3* KO, and 1,938 *N*-glycoforms were commonly found in both groups (**Figure 5B**). Our analysis yielded the first *N*-glycoproteomic dataset of immune cells from C57BL/6 mice.

Subsequently, we performed differential glycoform abundance analysis, and 180 *N*-glycoforms showed significant changes (|log2FC| ≥ 2, *p* < 0.05) in KO samples compared to WT samples; of these, 32 *N*-glycoforms (from 25 glycoproteins) increased in abundance and 148 *N*-glycoforms (from 104 glycoproteins) decreased in abundance (**Figure 5C**). As CD8^+^ T cell depletion experiments (**Figure 2**) suggested that the suppression of tumor growth in *B4galt3* KO mice may be caused by CD8^+^ T cells, we picked up T cell-associated GO entries and identified 14 proteins (PSAP, MMP9, MPO, HP, ITGAL, CTSZ, CD86, ITGA4, RPN2, PLD4, H2-AA, PIK3R1, ATP1B3, NGP) after GO analysis of the proteins corresponding to these glycoforms (**Figure 5D**, **Supplementary Table 8**). And because in the RNA-seq analysis of tumor-infiltrating CD8^+^ T cells, *B4galt3* deficiency was positively associated with integrin-mediated cell adhesion (**Supplementary Figure 3D**). Therefore, we focused on integrin alpha-L (ITGAL), which is expressed on the T cell surface and influences T cell activity within the TIME. We noted a considerable decrease in ITGAL-1 (aa183-200) and ITGAL-2 (aa925-938) glycoforms in *B4galt3* KO mice compared to their WT counterparts (**Figure 5E**). Both *N*-glycoforms of ITGAL were robustly corroborated by product ion spectra (**Supplementary Figures 5A, B**). Characteristic oxonium ions were annotated on the spectra, encompassing *m*/z values at 204.09 (HexNAc), 274.09 (NeuAc) and 290.09 (NeuGc), 366.14 (HexNAc + Hex), and 528.19 (Man + HexNAc + Hex). The ions corresponding to the peptides and peptide-associated fragments that were accurately interpreted constituted a substantial proportion of the total spectral intensity.

We used FACS to measure ITGAL protein expression on the surface of activated CD8^+^ T cells and confirmed that there was no change in ITGAL protein expression between KO and WT CD8^+^ T cells (**Figure 5F**), indicating that ITGAL glycoforms decreased because of glycosylation changes rather than total protein changes.

### 3.8 *B4galt3* KO enhances downstream phospho-FAK in ITGAL

**Figure 6:**
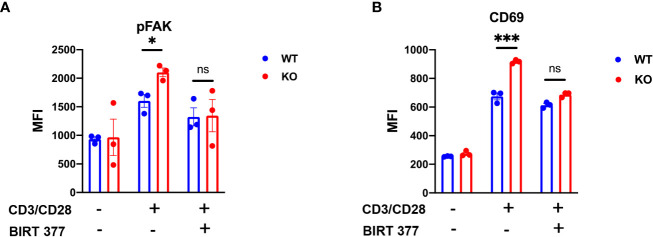
*B4galt3* KO enhances downstream pFAK in ITGAL. **(A)** Phosphorylated FAK of MFI, **(B)** CD69 MFI after 6-h stimulation with Dynabeads Mouse T-Activator CD3/CD28. Representative data from two independent experiments with 3 mice per experiment and group. Mean ± SEM, *p < 0.05, **p < 0.01, and ***p < 0.001; ns = not significant.

To elucidate the impact of decreased ITGAL glycoforms in CD8^+^ T cells on downstream signaling, we isolated CD8^+^ T cells from mouse spleens and stimulated them for 6-h. FACS analysis revealed that compared to WT CD8^+^ T cells, *B4galt3* KO CD8^+^ T cells displayed elevated levels of FAK phosphorylation. This difference in FAK phosphorylation was negated by the LFA-1-specific inhibitor BIRT377 (**Figure 6A**). Concurrently, KO CD8^+^ T cells expressed significantly higher levels of the T-cell activation marker CD69 (**Figure 6B**), suggesting increased ease of activation in *B4galt3* deficient CD8^+^ T cells.

## 4 Discussion

Over the past few decades, we have resolutely performed functional analysis of the B4GALT family (26). We generated KO mice deficient in *B4galt1*, *B4galt2*, *B4galt5*, and both *B4galt5* and *B4galt6* genes (27–30). This study primarily focused on the protein galactosyltransferase genes *B4galt1*, *B4galt3*, and *B4galt4*, rather than *B4galt5* and *B4galt6* involved in glycolipid synthesis. Our results showed that the growth of strongly immunogenic tumors was suppressed in *B4galt3* KO mice but not in *B4galt1* or *B4galt4* KO mice. To the best of our knowledge, this phenotype has not been reported in any other galactosyltransferase-KO mice. Notably, neither *B4galt1* nor *B4gal4* KO mice exhibited such phenotypes. This is likely due to the specificity of each galactosyltransferase in the glycosylation of different proteins, which could result in distinct physiological impacts. Each B4GALT member may influence distinct pathways involved in tumor progression or the immune response, resulting in different phenotypes. Furthermore, the expression level, activity as a galactosyltransferase, and tissue distribution of B4GALT3 may differ from those of B4GALT1 and B4GALT4, further contributing to the observed variations. To understand these differences, more comprehensive studies are required to investigate the molecular mechanisms and roles of B4GALT in tumor development and immunity.

Recent studies have shown that B4GALT3 is associated with glioblastoma, colorectal cancer, cervical cancer, bladder cancer, and lung metastasis with liver cancer (21, 22, 31–33). Furthermore, analysis of the expression of human *B4GALT3* mRNA in TCGA database revealed that *B4GALT3* was differentially expressed between tumor tissues and normal tissues, and the expression of *B4GALT3* was related to the prognosis of ACC, CESC, LIHC, MESO, SARC, and HNSC. Therefore, B4GALT3 plays an important role in tumorigenesis in humans. However, whether B4GALT3 is important in cancer or immune cells cannot be determined from these findings.

Owing to the selective suppression of growth in the highly immunogenic tumors observed in *B4galt3* KO mice, we explored the role of immune cells. We performed BM transplantation experiments to examine whether immune cells from the BM played a role in tumor growth suppression in *B4galt3* KO mice. WT mice transplanted with *B4galt3* KO BM cells, but not WT BM cells, showed suppressed tumor growth, indicating that immune cells are crucial. The TIME has various kinds of immune cells, among which CD8^+^ T cells play a key role in the anti-tumor function. After CD8^+^ T cells in mice were depleted using an anti-CD8 antibody, *B4galt3* KO mice did not respond to the growth inhibition of tumors with strong immunogenicity, demonstrating that CD8^+^ T cells are essential for suppressing tumor growth by *B4galt3* deficiency. Concurrently, the upregulation of genes associated with immune system processes, T cell differentiation, and interferon-gamma production were observed. Additionally, pathways associated with cell adhesion, T-cell receptor signaling, C-type lectin receptor signaling, and NF-κB were positively correlated with *B4galt3* deficiency.

We observed decreased infiltration of Tregs in the *B4galt3* KO TIME. An elevated number of CD8^+^ T cells in the TIME is typically associated with a favorable prognosis, whereas a high presence of Tregs is associated with a poor prognosis across numerous cancer types (34–37). CD8^+^ T cells inhibit tumor growth by secreting cytokines, such as interferon (IFN)-γ, granzyme B, and tumor necrosis factor-α (TNFα) (38). Our findings revealed that the TIME of *B4galt3* KO mice had a higher infiltration of IFN-γ^+^ and granzyme B^+^ CD8^+^ T cells than that of WT mice. IFN-γ and granzyme B are effective mechanisms through which CD8^+^ T cells kill tumor cells, which could directly contribute to the suppression of highly immunogenic tumor growth. These findings suggest the formation of a tumor-suppressive microenvironment in *B4galt3* KO mice.

To characterize WT and *B4galt3* KO CD8^+^ T cells in the TIME, we used FACS to isolate CD8^+^ T cells and performed RNA sequencing. GO analyses showed that the regulation of cell proliferation and inflammatory response-related genes increased. The inflammatory responses of T cells are associated with an increase in their anti-tumor effects. By increasing the inflammatory response of T cells, the immune system is better able to recognize and attack cancer cells. Recent studies report that B4GALT3 regulates the ITGB1 (20–22). Although not statistically significant, we found that *B4galt3* deficiency was positively correlated with the integrin-mediated cell adhesion pathway.

Through glycoproteomic analyses, we specifically identified the ITGAL, which is part of the integrin family and associated with T cell activation. Integrins are among the family of molecules whose functions change owing to glycosylation. Integrin family members are key adhesion molecules for recruiting immune cells to the site where an immune response is taking place. Particularly, ITGAL binds to ITGB2 to generate LFA-1. LFA-1 is a mechanosensitive adhesion receptor that uses mechanical forces to regulate T cell migration, differentiation, and effector functions (39). Activating LFA-1 on tumor-infiltrating T cells to boost its binding to ICAM-1 could potentially increase tumor-specific T cell recruitment to the TIME (40). The elevated FAK phosphorylation and increased expression of T cell activation marker CD69 in *B4galt3* KO CD8^+^ T cells suggests that B4GALT3 may play a role in regulating T cell activation. FAK is a key player in integrin-mediated signal transduction pathways and its phosphorylation is an indicator of cell activation. The increase in FAK phosphorylation in *B4galt3* KO T cells could therefore reflect enhanced T cell activation, potentially contributing to the increased anti-tumor activity in *B4galt3* KO mice. Certain research findings have demonstrated that the conformation of LFA-1 plays a crucial role in modulating T cell activity and cytotoxicity (41, 42). Changes in the glycosylation of ITGAL could potentially alter the extension of LFA-1 conformation. Altered conformations may enhance ITGAL’s interactions with ligands or other molecules, amplifying downstream signaling pathways like FAK phosphorylation. This could enhance the migratory and infiltrative capabilities of T cells, facilitating greater CD8^+^ T cell infiltration into tumors and inhibiting tumor growth. While it’s possible that altered glycosylation affects ITGAL stability, our data suggest that the changes more likely influence ITGAL-mediated signaling, as ITGAL expression levels were comparable between WT and KO after *in vitro* stimulation (**Figure 5F**). However, direct evidence supporting this hypothesis is currently lacking, underscoring the need for further research.

The elevated expression of B4GALT3 in multiple tumor types, including ACC, CESC, LIHC, MESO, SARC, and HNSC, underscores its potential as a biomarker for these cancers. Building on this, our study with *B4galt3* KO mice has revealed new opportunities for therapeutic interventions. Specifically, the use of siRNA to inhibit B4GALT3 expression emerges as a promising strategy for modulating glycosylation pathways. Our research has particularly highlighted the role of ITGAL in modulating CD8^+^ T cell activity through glycosylation, opening avenues for the development of targeted therapies to enhance anti-tumor immunity. Importantly, our *B4galt3* KO mice showed no observable health defects over a year-long observation period, supporting the idea that targeting B4GALT3 could be a therapeutic approach with a favorable safety profile.

In conclusion, our study provides new insights into the role of B4GALT3 in mediating tumor-immune interactions. We demonstrated that *B4galt3* deficiency affects the TIME, leading to increased infiltration of CD8^+^ T cells, thereby inhibiting the growth of strongly immunogenic tumors. Interestingly, our glycoproteomic analysis suggests a potential association between *B4galt3* deficiency and alterations in glycosylation of the ITGAL protein on the surface of T cells. Nevertheless, our findings present new therapeutic possibilities, particularly for tumors with high immunogenicity. Furthermore, the *B4galt3* KO mouse generated in our study offers a valuable tool for future research on the complex interactions between glycosylation and immune responses in cancer.

## Data availability statement

The datasets generated and/or analyzed during the current study, as well as the online tools used, are as follows: RNA-seq data of tumor tissues were deposited in the NCBI SRA repository under accession number PRJNA856497. RNA-seq data of tumor-infiltrating CD8^+^ T cells are also available at the NCBI SRA repository under accession number PRJNA982840. Raw data files pertaining to *N*-glycoproteomic analysis were lodged within the ProteomeXchange Consortium *via* the jPOST partner repository, bearing the dataset identifier PXD041293. The flow cytometry raw results can be accessed *via* https://flowrepository.org/id/, identification number FR-FCM-Z6M6, FR-FCM-Z6M7, FR-FCM-Z6M8, FR-FCM-Z6M9, FR-FCM-Z6MA, FR-FCM-Z6MB, FR-FCM-Z6MC, and FR-FCM-Z6MD. In addition to the datasets, the study also used the following online resources: The Konezumi online tool, accessible at: (https://www.md.tsukuba.ac.jp/LabAnimalResCNT/KOanimals/konezumi.html). The TIMER2.0 database, accessible at: (http://timer.cistrome.org). The GEPIA2 online tool, accessible at: (http://gepia2.cancer-pku.cn/#index).

## Ethics statement

Ethical approval was not required for the study involving humans in accordance with the local legislation and institutional requirements. Written informed consent to participate in this study was not required from the participants or the participants’ legal guardians/next of kin in accordance with the national legislation and the institutional requirements. The animal study was approved by Animal Experimentation Committee of Kyoto University. The study was conducted in accordance with the local legislation and institutional requirements.

## Author contributions

HW: Investigation, Resources, Software, Visualization, Writing – original draft, Writing – review & editing. CN: Project administration, Supervision, Writing – original draft, Writing – review & editing. DT: Investigation, Software, Visualization, Data curation, Methodology, Writing – original draft, Writing – review & editing. KS: Resources, Writing – review & editing. XP: Writing – review & editing. AI: Resources, Writing – review & editing. NK: Project administration, Resources, Supervision, Writing – original draft, Writing – review & editing. MA: Project administration, Supervision, Writing – original draft, Writing – review & editing.
