# Supplementary material for: Beta-1,4-galactosyltransferase-3 deficiency suppresses the growth of immunogenic tumors in mice
Source: Front Immunol. 2023 Oct 9;14:1272537. doi: 10.3389/fimmu.2023.1272537 (PMC10600447; doi:10.3389/fimmu.2023.1272537)
Supplement: Supplementary file 1 [file DataSheet_1.pdf]

Supplementary Figure 1

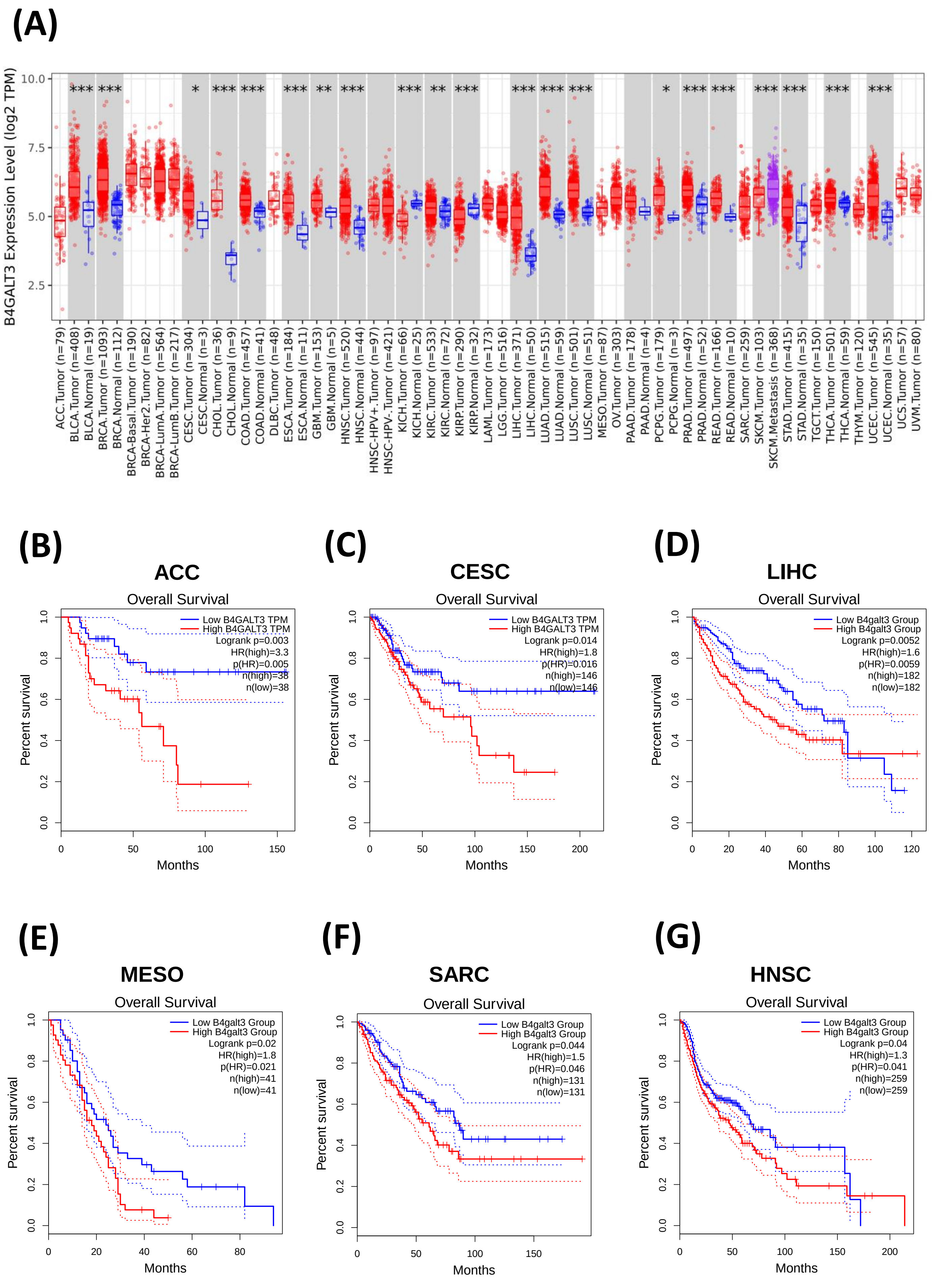

Supplementary Figure 1. Messenger RNA expression levels of human *B4GALT3* gene in pan-cancers. (A) Human *B4GALT3* expression levels in different cancer types from TCGA data in TIMER. *$p < 0.05$, **$p < 0.01$, ***$p < 0.001$. (B–G) *B4GALT3* expression and the prognosis of patients with adenoid cystic carcinoma (ACC), cervical squamous cell carcinoma and endo-cervical adenocarcinoma (CESC), liver hepatocellular carcinoma (LIHC), mesothelioma (MESO), sarcoma (SARC), head and neck squamous cell carcinoma (HNSC) in the TCGA using GEPIA2 online tool.

Supplementary Figure 2

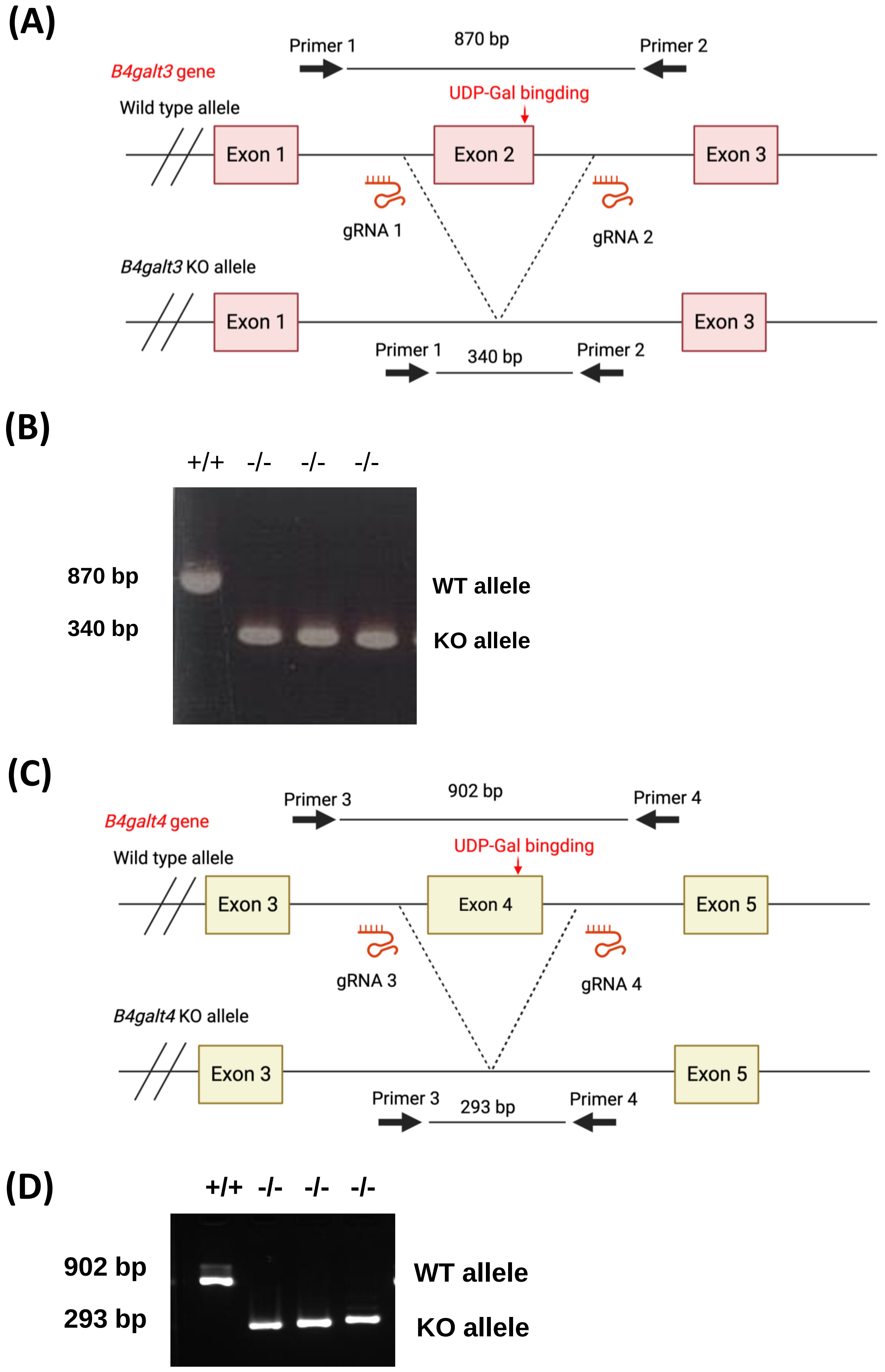

Supplementary Figure 2. *B4galt3* and *B4galt4* KO strategy and genotyping. (A) *B4galt3* KO strategy using the CRISPR/Cas9 system. Guide RNA 1 and 2 were set at the upstream and downstream of the exon 2 to delete the exon 2 containing UDP-Gal binding site. (B) Genotyping was performed by PCR analysis using primer 1 and primer 2. The PCR product for the WT allele is 870 bp (upper), whereas that for the KO allele is 340 bp (lower). (C) *B4galt4* KO strategy using the CRISPR/Cas9 system. Guide RNA 3 and 4 were set at the upstream and downstream of the exon 4 to delete the exon 4 containing UDP-Gal binding site. (D) Genotyping was performed by PCR analysis using primer 3 and primer 4. The PCR product for the WT allele is 902 bp (upper), whereas that for the KO allele is 293 bp (lower).

Supplementary Figure 3

(A)

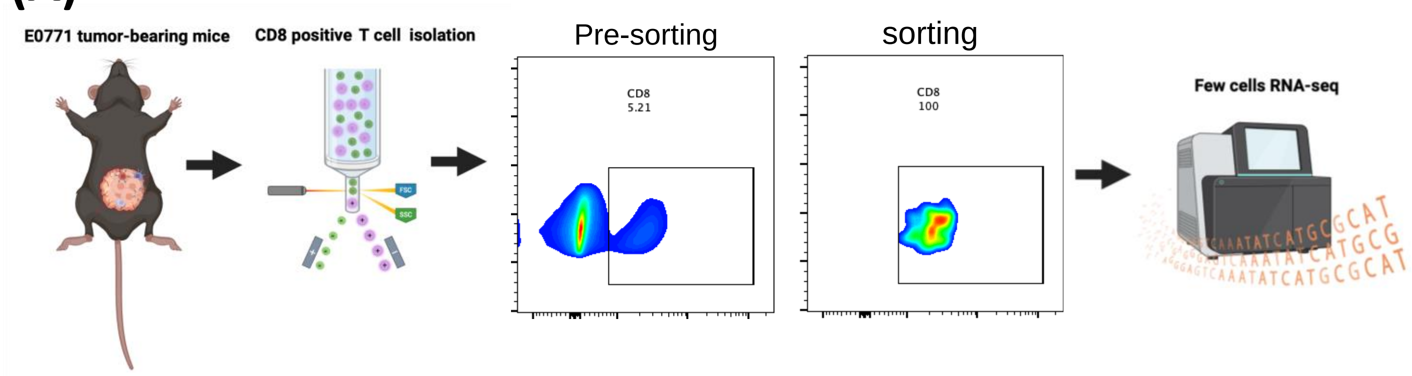

(B)

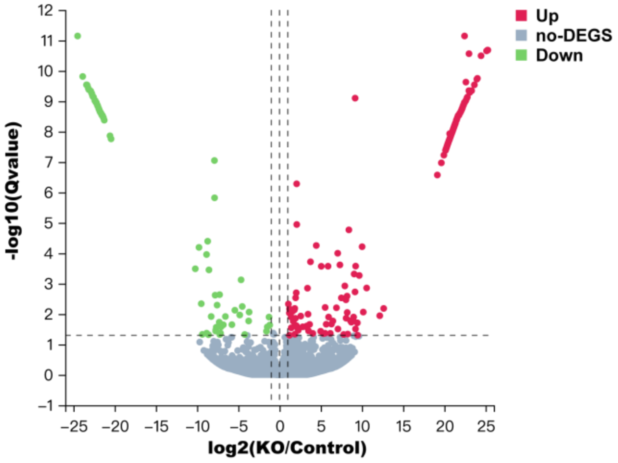

(C)

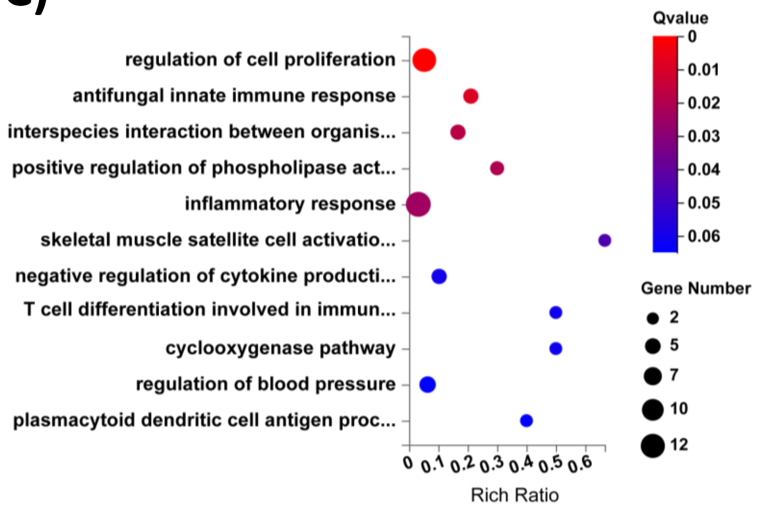

(D)

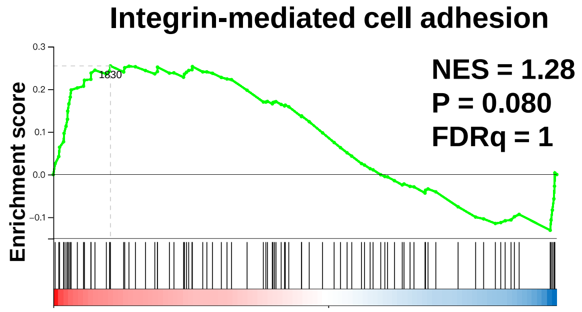

Supplementary Figure 3. Gene expression characteristics of tumor-infiltrating $CD8^+$ T cells. (A) Schematic of RNA-seq analysis of tumor-infiltrating $CD8^+$ T cells. (B) DEGs were defined as the genes with $|\log2FC| \geq 1$ and $FDR < 0.05$. Red dots represent up-regulated genes, and green dots represent down-regulated genes in *B4galt3* KO mice compared to WT mice. WT, n = 4; KO, n = 5. (C) GO enrichment biological process analysis of up-regulated genes in *B4galt3* KO mice compared to WT mice. (D) GSEA of the genes associated with Integrin-mediated cell adhesion. NES, normalized enrichment score; FDR, false discovery rate. All analyses were performed by comparing the *B4galt3* KO against WT counterparts, which served as the control group.

Supplementary Figure 4

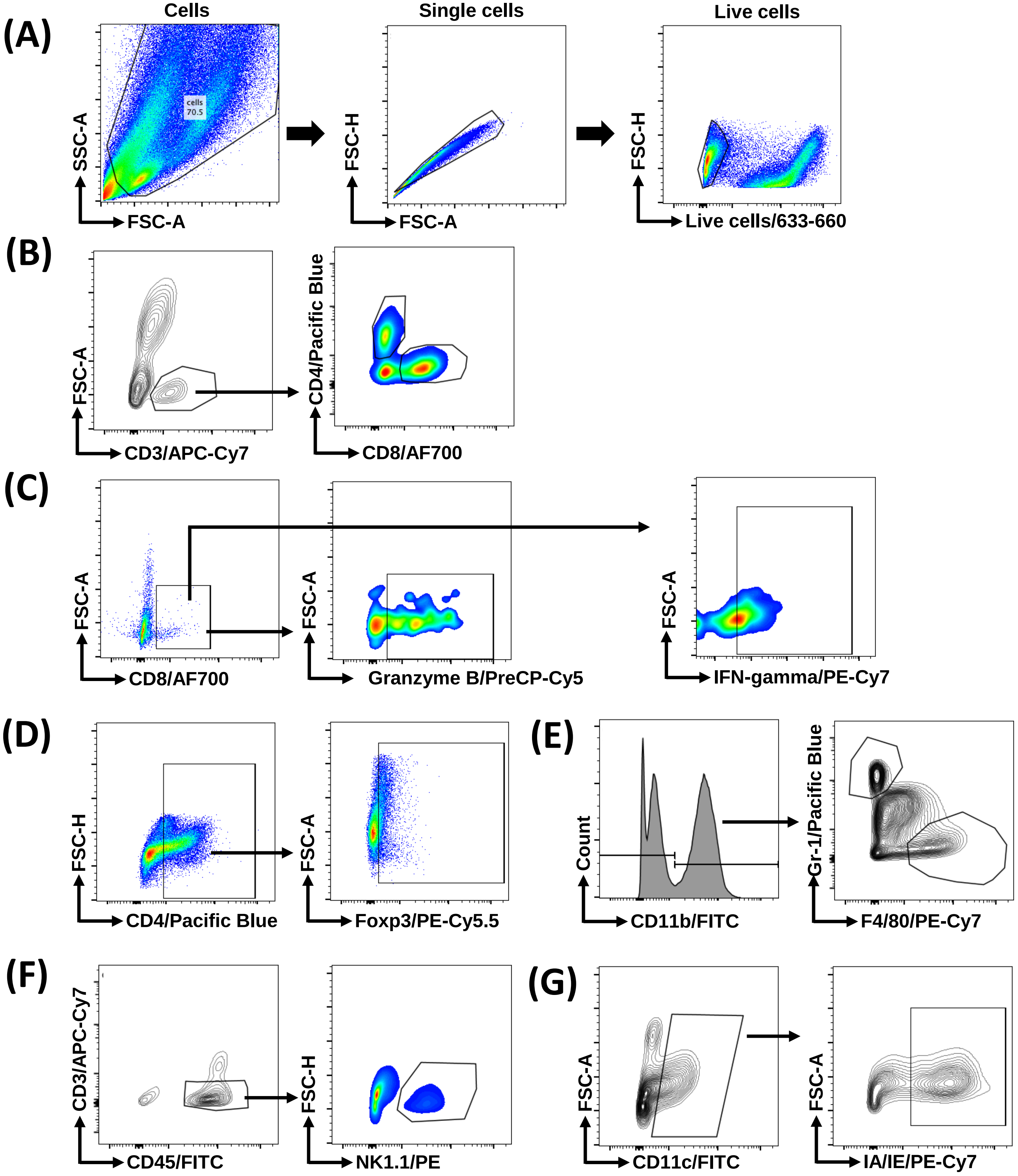

Supplementary Figure 4. Gating strategy for flow cytometry analysis of the TIME. (A) The figure depicts the gating strategies for identifying live cells. (B) CD3-positive T cells, CD8-positive T cells, CD4-positive T cells. (C) CD8-positive T cells, granzyme B-positive cells and IFN-γ-positive cells. (D) CD4-positive T cells, Foxp3-positive cells. (E) CD11b-positive cells, Gr-1-positive cells (MDSC: myeloid-derived suppressor cells), and F4/80-positive cells (macrophages). (F) CD45-positive CD3-negative cells and NK1.1-positive cells (NK cells). (G) CD11c-positive cells and IA/IE-positive cells (dendritic cells).

Supplementary Figure 5

(A)

ITGAL-1 (aa183-200)

**Integrin alpha-L aa183–200**	Precursor ion: 1120.9532 (z=4)<br>Glycan composition: $HexNAc_4Hex_5Fuc_1NeuGc_2$

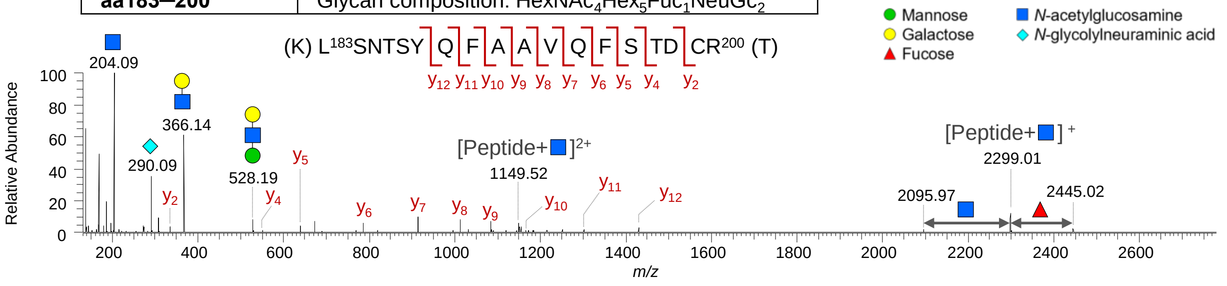

(B)

ITGAL-2 (aa925-938)

**Integrin alpha-L aa925–938**	Precursor ion: 1294.2057 (z=3)<br>Glycan composition: $HexNAc_4Hex_6Fuc_1NeuAc_1$

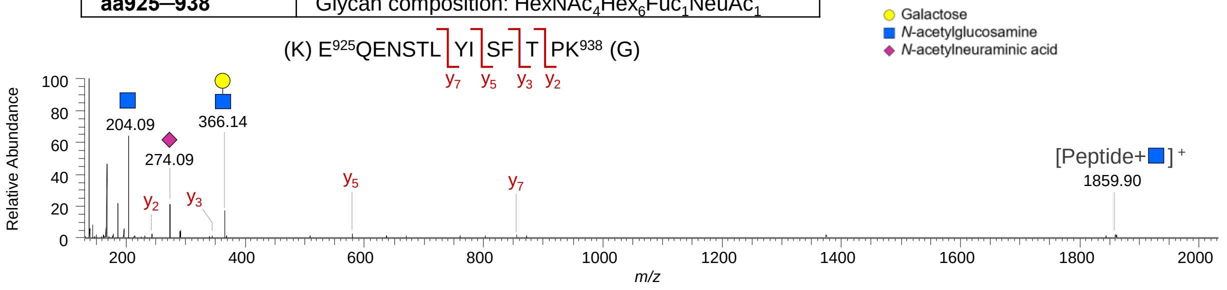

Supplementary Figure 5. Spectra of product ions originating from *N*-glycopeptides. (A) ITGAL-1 (aa183-200). (B) ITGAL-2 (aa925-938). Symbols: green circle, mannose; yellow circle, galactose; red triangle fucose; blue square, *N*-acetylglucosamine; cyan diamond, *N*-glycolylneuraminic acid; purple diamond, *N*-acetylneuraminic acid.
