# Supplementary material for: Beta-1,4-galactosyltransferase-3 deficiency suppresses the growth of immunogenic tumors in mice
Source: Front Immunol. 2023 Oct 9;14:1272537. doi: 10.3389/fimmu.2023.1272537 (PMC10600447; doi:10.3389/fimmu.2023.1272537)
Supplement: Supplementary file 2 [file Table_1.docx]

**Supplementary Table 1.** gRNA sequences and primers used in gene editing and genotypic verification.

*B4galt3* gRNA sequences

| gRNA 1 | AAGGCAAGGTCAGAAATCGG |
| --- | --- |
| gRNA 2 | GTGGGGTAACTGTAAGACAG |

*B4galt4* gRNA sequences

| gRNA 3 | CATTGTGCCTCATCGTGCCC |
| --- | --- |
| gRNA 4 | CATCGTGCCCGGGAGCACCA |

*B4galt3*-genoryping primers sequences

| Primer 1 | CCCTAGGGTGGGAATCATTT |
| --- | --- |
| Primer 2 | TTTGTGTACGAGCGTTTTGC |

*B4galt4*-genoryping primers sequences

| Primer 3 | AGCATTTGGGAGTCTGAGGC |
| --- | --- |
| Primer 4 | ATTGGACGGGTAGGTCGAAC |
