# Supplementary material for: Beta-1,4-galactosyltransferase-3 deficiency suppresses the growth of immunogenic tumors in mice
Source: Front Immunol. 2023 Oct 9;14:1272537. doi: 10.3389/fimmu.2023.1272537 (PMC10600447; doi:10.3389/fimmu.2023.1272537)
Supplement: Supplementary file 3 [file Table_2.docx]

| Cell line | Source | Culture conditions | Number of transplants |
| --- | --- | --- | --- |
| B16F0 | RIKEN BioResource Center  （Ibaraki, Japan） | RPMI-1640 containing, Glutamax (Gibco), 10% fetal bovine serum(Serana), and 1% penicillin/streptomycin (Wako). | 1 × 10^5^ cells |
| B16F10 | RIKEN BioResource Center  （Ibaraki, Japan） | RPMI-1640 containing, Glutamax (Gibco), 10% fetal bovine serum (Serana), and 1% penicillin/streptomycin (Wako). | 1 × 10^6^ cells |
| B16F10-OVA | Dr. Yoshihiro Hayakawa (University of Toyama, Japan) | RPMI-1640 containing, Glutamax (Gibco), 10% fetal bovine serum(Serana), and 1% penicillin/streptomycin (Wako). | 1 × 10^6^ cells |
| MB49 | Merck Sigma-Aldrich | High-glucose DMEM (Wako) containing, Glutamax (Gibco), MEM non-essential amino acids (Gibco), 1 mM sodium pyruvate (Gibco), 10% fetal bovine serum (Serana), and 1% penicillin/streptomycin (Wako). | 2 × 10^5^ cells |
| EO771 | ATCC | High-glucose DMEM (Wako) containing, Glutamax (Gibco), MEM non-essential amino acids (Gibco), 10% fetal bovine serum (Serana), 1% penicillin/streptomycin (Wako) and 20mM HEPES (Gibco). | 1 × 10^6^ cells |
| MC38 | Kerafast (Boston, MA, USA). | High-glucose DMEM (Wako) containing, Glutamax (Gibco), MEM non-essential amino acids (Gibco), 1 mM sodium pyruvate (Gibco), 10% fetal bovine serum (Serana), and 1% penicillin/streptomycin (Wako). | 1 × 10^6^ cells |

**Supplementary Table 2.** Cell Lines, sources, culture conditions, and transplant quantities.
